# HuR-Regulated mRNAs Associated with Nuclear hnRNP A1-RNP Complexes

**DOI:** 10.3390/ijms141020256

**Published:** 2013-10-11

**Authors:** Olga Papadodima, Aristotelis Chatziioannou, Meropi Patrinou-Georgoula, Fragiskos N. Kolisis, Vasiliki Pletsa, Apostolia Guialis

**Affiliations:** 1 Division of Biological Research and Biotechnology, Institute of Biology, Medicinal Chemistry and Biotechnology, National Hellenic Research Foundation, 48 Vas. Constantinou Avenue, Athens 11635, Greece; E-Mails: opapadod@eie.gr (O.P.); achatzi@eie.gr (A.C.); mpatr7@gmail.com (M.P.-G.); 2 Laboratory of Biotechnology, School of Chemical Engineering, National Technical University of Athens, Athens 15780, Greece; E-Mail: kolisis@chemeng.ntua.gr

**Keywords:** RNA-binding proteins (RBPs), ribonucleoprotein (hnRNP) complexes, post-transcriptional regulation, mRNA processing, RNA-immunoprecipitation (RIP)-Chip technology, mouse embryonic fibroblasts (MEFs)

## Abstract

Post-transcriptional regulatory networks are dependent on the interplay of many RNA-binding proteins having a major role in mRNA processing events in mammals. We have been interested in the concerted action of the two RNA-binding proteins hnRNP A1 and HuR, both stable components of immunoselected hnRNP complexes and having a major nuclear localization. Specifically, we present here the application of the RNA-immunoprecipitation (RIP)-Chip technology to identify a population of nuclear transcripts associated with hnRNP A1-RNPs as isolated from the nuclear extract of either HuR WT or HuR-depleted (KO) mouse embryonic fibroblast (MEF) cells. The outcome of this analysis was a list of target genes regulated via HuR for their association (either increased or reduced) with the nuclear hnRNP A1-RNP complexes. Real time PCR analysis was applied to validate a selected number of nuclear mRNA transcripts, as well as to identify pre-spliced transcripts (in addition to their mature mRNA counterpart) within the isolated nuclear hnRNP A1-RNPs. The differentially enriched mRNAs were found to belong to GO categories relevant to biological processes anticipated for hnRNP A1 and HuR (such as transport, transcription, translation, apoptosis and cell cycle) indicating their concerted function in mRNA metabolism.

## 1. Introduction

RNA-binding proteins (RBPs) that associate with RNA pol II transcripts (pre-mRNA or hnRNA) constitute a large group of cellular proteins that are key components of macromolecular assemblies functioning in post-transcriptional events such as splicing, polyadenylation, transport, localization and stability/translation of mRNA. This is brought about by the extensive interplay amongst discrete sets of RBPs and their associated (pre)- and mRNA-target molecules in the form of ribonucleoprotein (RNP) complexes (reviewed in [1,2]).

Heterogeneous nuclear ribonucleoproteins (hnRNPs) are a major group of mostly nuclear RBPs in dynamic association with nascent hnRNA (pre-mRNA) and processed mRNA in the form of hnRNP complexes [3,4]. Likewise, cytoplasmic mRNP complexes contain mature mRNA bound to a discrete group of RBPs, including some exported hnRNP protein species [5]. Other important RNA-protein assemblies participating in mRNA processing refer to the spliceosomal small nuclear RNPs (U1, U2, U4/U6 and U5 snRNP) [6], as well as to complexes of microRNAs (miRNAs) with their associated proteins functioning in translational control [7].

The hnRNP proteins comprise a group of over 20 polypeptides in the range of 32 to 110 kDa and designated in order of increasing molecular weight as hnRNP A-U [8,9]. They are abundant nuclear proteins containing at least one motif for binding to RNA (the RBD/RRM, KH or RGG domains). Almost all hnRNP proteins contain multiple isoform types, products of alternative splicing, as well as post-translational modifications (phosphorylation, arg-methylation). As they form a network of interactions with the RNA and with other protein components, they are considered important gene regulators functioning, in addition to mRNA slicing (mainly alternative splicing), in almost every step of mRNA biogenesis, and in many other cellular functions [4]. The most abundant members of hnRNPs are those of the hnRNP A/B type (mainly hnRNPA1 and A2/B1), with hnRNP A1 considered a prototype hnRNP protein [10]. HnRNP A1, like most hnRNPs, binds to nascent transcripts and remains associated with the target RNAs through all mRNA processing steps in the nucleus. Due to its ability for nucleo-cytoplasmic shuttling, it accompanies mature mRNA through transport to the cytoplasm and association with the translated polysomes [11,12].

An additional RNA-binding protein of importance to mRNA maturation processes is HuR, a ubiquitously expressed and best characterized member of the small family of embryonic lethal abnormal vision (ELAV) Hu proteins (HuR, HuB, HuC and HuD) [13]. Like most hnRNP proteins, HuR has a major nuclear localization with ability for nucleo-cytoplasmic shuttling [14]. HuR is the prototype of a group of RBPs that bind AU-rich elements (AREs) commonly found in the 3′-untranslated region (UTR) of short-lived mRNAs (like proto-oncogenes, cytokines, and lymphokines). AREs regulate stability, translation and localization of the ARE-containing mRNAs upon application of stimuli like cell activation, malignant transformation and exogenous stress [15,16]. HuR is found to promote stabilization of its target mRNAs [14], opposite to other ARE-binding proteins (e.g., TIA-1 and TIAR) that cause destabilization and degradation of the bound mRNAs [17]. Several members of the hnRNPs, such as hnRNP A1, D/AUF1 and L, are also ARE-binding proteins that promote decay of bound mRNAs and antagonize the role of HuR [18].

Despite the early recognition that under normal cellular growth the bulk (over 90%) of HuR is in the nucleus [19,20], it was mostly known for its cytoplasmic functioning in stability/translation of target mRNAs. It is only recently that HuR’s participation in nuclear events has been recognized [13]. An increasing number of reports indicate the active involvement of HuR in nuclear mRNA processing events (splicing and polyadenylation) and in export of target mRNAs to the cytoplasm [21,22]. Connected to its multi-faceted role in post-transcriptional events, HuR has an important role in cellular physiology as it regulates many cellular processes, including cell cycle, differentiation, apoptosis and DNA damage response [23,24].

A breakthrough in the study of the assemblies of cellular mRNAs with their RNA-binding proteins (referred to as the Ribonome) was the application of the Ribonomics platform by Keene’ lab [25]. This provided the ground to study, at genome-wide level, the mRNA transcript population associated with a specific RBP, ascribed to a particular cell type and growth state. The Ribonomics platform (RIP-Chip assay) refers to Ribonucleoprotein (RNP) ImmunoPrecipitation (RIP) followed by microarray (Chip) analysis. The first step (RIP assay) is the application on a cell extract of an antibody targeting a selected RNA-binding protein and subsequent RNA isolation from the immune pellets. In the second step (Chip analysis) the microarray and computational analysis of the isolated RNAs is taking place. The RIP-Chip method has been applied to a constantly increasing number of RBP proteins by immunoselecting their respective RBP-RNP complexes isolated under conditions that preserve their integrity and allows identification of the kinetically stable RBP-RNP-mRNA population [26]. RBP-specific RNA transcripts have been also investigated, using high stringency conditions on the immunoselected RBP-RNP complexes to eliminate any associated proteins [26]. Advanced methods are currently applied to yield, in addition, the binding sites at nucleotide resolution of the target mRNAs. These refer to the Cross-Linking and ImmunoPrecipitation (CLIP) assays and the modified iCLIP method that provide information on the stably as well as transiently RBP protein interacting mRNAs [27–29]. A modified method named Photoactivatable ribonucleoside-enhanced CLIP (PAR-CLIP) is in use that also permits detection of the RBP-binding sites on the target mRNA [30].

The basis of the RIP-Chip application in the present study stems from our recent reports for the existence of a broad range of interactions between HuR and hnRNP proteins existing within endogenous hnRNP/mRNP, as well as HuR assemblies, as they are obtained from extracts of mammalian (human/mouse) cell origin [31,32]. Specifically, we relied on our findings for the ability of HuR to associate in an RNA-dependent manner with immunoselected hnRNP A1-RNP (and hnRNP C1/C2-RNPs) nuclear complexes, as well as with cytoplasmic hnRNP A1-associated mRNPs. As we have been interested in the nuclear role of HuR, we performed the current follow up study aiming at mapping the sub-population of mRNA transcripts that were stably associated with the nuclear hnRNP A1-RNP complexes in the presence or absence of co-precipitated HuR. The RIP-Chip platform was applied on HuR-containing and HuR-lacking hnRNP A1-RNP complexes, as obtained from the nuclear fractions of HuR Wild Type (WT) and HuR Knock-Out (KO) Mouse Embryonic Fibroblast (MEF) cells [33]. We present here our findings on the identification of RNA transcripts having a differential expression level in the nuclear RNA population and, subsequently, the hnRNP A1-RNP-bound targets for the HuR WT and HuR KO cells. The combined microarray and bioinformatics analysis also provided interesting Gene Ontology (GO) categories ascribed to the tested cell line. The application of Real Time quantitative (RT-q) PCR on selected mRNA targets assisted in validating the microarray data and, furthermore, in detecting the presence of spliced and pre-spliced mRNAs in the isolated hnRNP A1-RNP complexes. Through these findings, a novel definition of the HuR-dependent pattern of hnRNP A1-RNP-associated transcripts is provided.

## 2. Results

### 2.1. Ribonomic Profiling of hnRNP A1-RNP Complexes Isolated from Nuclear Extracts of HuR-Containing and HuR-Lacking MEF Cells

The general strategy applied in the present study was based on our previous identification of hnRNP A1 (as well as hnRNP C1/C2) as associating with HuR [31,32] and we focused on the identification, amongst the plethora of reported HuR targets, of those that were in stable association with hnRNP A1-RNP complexes pre-existing in the nuclear extracts of mammalian cell origin. To apply the Ribonomics approach, hnRNP A1 was the protein of choice as a prototype, abundant protein component of hnRNP complexes [34] sharing with HuR a major nuclear localization and ability to shuttle between the nuclear and cytoplasmic cellular compartments [11,12,14]. To this end, we selected the cellular system of cultured mouse embryonic fibroblast (MEF) cells by taking advantage of the availability of a cell derivative completely devoid of HuR (HuR KO) [33]. The use of paired MEF cells either HuR WT or HuR KO, thus, provided a suitable experimental system to compare within purified nuclear hnRNP complexes any co-selected RNA species. Nuclear extracts, prepared under conditions that prevent dissociation of endogenous hnRNP complexes, were subjected to RNA immunoprecipitation (RIP assay) using the monoclonal 4B10 antibody targeting hnRNP A1, which is known to co-precipitate with A1 all other stably associated hnRNP components, including any additional RBPs, like HuR [31,35]. The subsequent identification of co-selected RNAs (pre- and mRNA transcripts, and also any non-coding RNAs) will, thereafter, be considered as potential target RNAs associated with endogenous hnRNP A1-RNP complexes, in the presence or absence of co-precipitated HuR.

#### 2.1.1. RNA Immunoprecipitation (RIP) Assay

MEF nuclear extracts were first incubated with a mouse IgG2 fraction (same isotype of the 4B10 hnRNP A1 antibody) that served as control IP (IgG-co) and, then, with the equivalent amount of the 4B10 antibody. Triplicate experiments were performed on an equal number of HuR WT and HuR KO cells. The immunoselected nuclear hnRNP A1-RNPs and their respective IgG-control assays from either HuR WT or HuR KO cells, together with an aliquot of the nuclear extract taken prior to IP, were the basis of obtaining the RNA population for subsequent microarray analysis.

**Figure 1:**
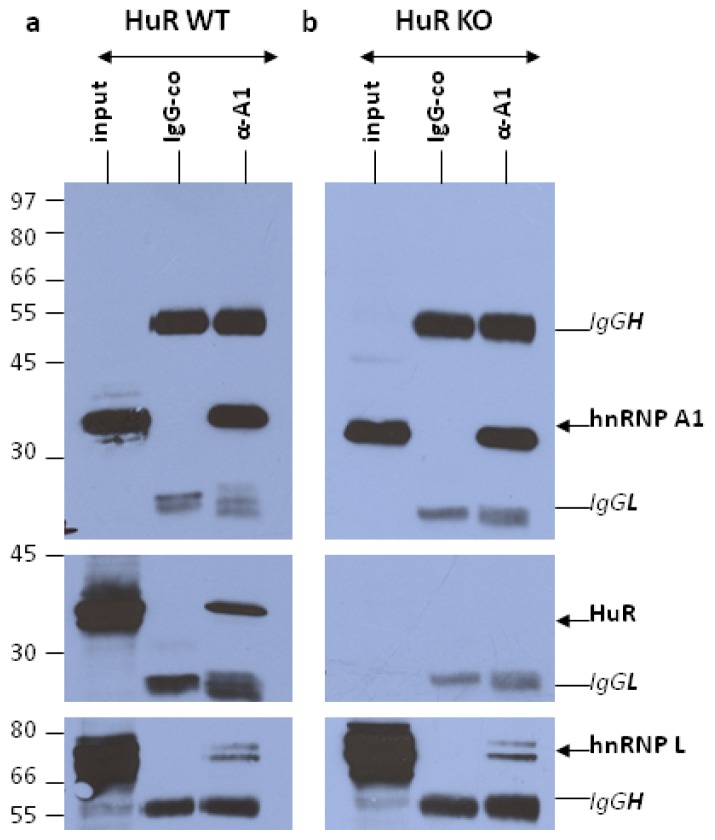
Verification of the specificity and efficiency of the anti-hnRNP A1 Immunoprecipitation (IP) reactions. IPs were performed on nuclear extracts obtained from both HuR WT (**a**) and HuR KO (**b**) MEFs by applying either the mouse monoclonal anti-hnRNP A1 (4B10) antibody or the mouse IgG2 purified immunoglobulin of same isotype as the anti-hnRNP A1 antibody that served as control IP (IgG-co). Proteins present in the input and immune pellets were resolved by SDS-PAGE and transferred to nitrocellulose. Western blotting was performed using mouse monoclonal antibodies against HuR, hnRNP A1 and L. The cross-reacting mouse IgG light (IgG_L_) and heavy (IgG_H_) chains are also marked. A representative picture referring to one of the triplicate IP assays is shown.

To test the specificity and efficiency of the anti-hnRNP A1 IP reactions, in direct comparison to the IgG-control assays, and to verify the presence or absence of co-precipitated HuR we applied western blotting analysis on an aliquot of the immunoselected nuclear RNP complexes. A representative picture referring to one of the triplicate IPs on HuR WT and HuR KO nuclear extracts is shown (Figure 1). In HuR WT cells hnRNP A1 was detected with high specificity and efficiency in the anti-hnRNP A1 pellets, alone, as was also the case of the co-selected HuR. As anticipated, in the HuR KO cells there was a complete lack of HuR in the nuclear extract and, therefore, in the corresponding hnRNP A1-RNP complexes. Despite the lack of quantitative estimates, a gross comparison of input and precipitated (α-A1) protein levels, made within the same western blot, indicated roughly comparable amounts of hnRNP A1 in the nuclear extract and the immune pellets alike, in HuR WT and HuR KO extracts. This appeared to be the case of another major protein of hnRNP complexes; the hnRNP L.

The RNA-extraction protocol was applied to all immune pellets (hnRNP A1-RNP and IgG-control assays) of HuR WT and HuR KO cells to obtain any tightly associated RNA transcripts, as well as to the nuclear extracts for the total nuclear RNA. Based on the estimated amount (OD_260_) of the isolated RNA from the triplicate experiments, the percentage (about 8%) of RNA isolated from the hnRNP A1-RNP pellets relative to their respective total RNA was similar between HuR WT and HuR KO cells. Contrary, a much lower amount of RNA (much below 0.2%) was obtained from the IgG-control nuclear pellets, indicative of a highly specific IP reaction with practically undetected RNA background levels. This too low amount of isolated nuclear IgG-control RNA (alike in HuR WT and HuR KO cell cultures) could not meet the requirement of microarray analysis and was, thereafter, excluded from any further analysis. Instead, we relied on the total nuclear RNA expression levels in HuR WT and HuR KO cells in order to estimate the relative enrichment of the RNA transcripts within the immunoselected hnRNP A1-RNP complexes.

#### 2.1.2. Microarray (Chip) Analysis

The isolated total nuclear RNA along with the hnRNP A1-RNP-associated RNA transcripts from either HuR WT or HuR KO cells were subjected to microarray analysis by the application of the Illumina MouseWG-6 platform combined with bioinformatics.

We first compared the expression level of the detected total nuclear RNA transcripts between HuR KO and HuR WT cells by statistical analysis coupled to fold change filtering. In particular, *t*-test was performed (*p*-value < 0.05 and FDR < 0.05) and after setting a baseline of two-fold change in their expression level in KO *vs*. WT cells, we obtained a number of 529 differentially expressed transcripts, which are presented in the Table S1. This list of transcripts contained 261 up-regulated and 268 down-regulated RNAs. In Table 1 the priority lists of most up- and down-regulated genes in the nuclear extract of HuR KO/HuR WT cells are shown.

The differentially expressed RNA transcripts in the total nuclear RNA population in HuR WT and KO cells were subjected to Gene Ontology (GO) analysis on the basis of Biological Processes (BPs); a restricted number of BPs were likely to be affected by the HuR’s absence. More specifically, in the case of the up-regulated transcripts the prominent GOs referred to regulation of transcription, cell cycle and oxidation-reduction while discrete GO categories were ascribed for the down-regulated transcripts, affecting BPs like development, transport and apoptosis.

We then proceeded to the identification of RNA transcripts associated with the hnRNP A1-RNP complexes in nuclear extracts of either HuR KO or HuR WT cells, aiming to identify enriched transcripts in the IP reactions, as compared to the input RNA (*t*-test: *p*-value < 0.05 and FDR < 0.05). To assign any of these transcripts as true target genes of the immunopurified complexes, we considered that their enrichment, calculated as the relative abundance in the IP reaction compared to the corresponding expression level in total nuclear RNA, should be above 2. In this way, we have directly related the association of the hnRNP A1-RNP transcripts to their expression level in the total nuclear RNA population. As anticipated, Gapdh mRNA, serving as an abundant mRNA and a reported non-target of HuR and of hnRNP A1-RNP complexes [17,36] was identified as a low contaminant (<2 fold) in our isolated hnRNP A1-RNPs, in HuR KO and WT cells alike.

Following the application of a high stringency enrichment score (IP *vs*. total nuclear RNA > 2), we obtained 601 nuclear transcripts stably associated with HuR WT hnRNP A1-RNPs and 731 transcripts in the HuR KO complexes. These numbers corresponded to 2.1% and 2.4% of the total genes on the IlluminaBeadChip platform for HuR WT and HuR KO cell types, respectively. The targets with the highest enrichment scores depicted an up to 5-fold increase in the IP reaction as compared to total RNA. Moreover, as seen in Table 2, a larger number of hnRNP A1-RNP-associated transcripts in the high range of 4 to 5-fold change were seen in the case of HuR KO compared to HuR WT RNP complexes (22 and 3 transcripts, respectively).

### 2.2. Differential Association of Target RNA Transcripts with hnRNP A1-RNP Complexes in Nuclear Extracts of HuR-Containing and HuR-Lacking MEFs

Thus far, the microarray data analysis provided a list of hnRNP A1 targets in HuR-containing (WT) and HuR-lacking (KO) nuclear RNP complexes. This allowed us to proceed to estimates of their differential association in the two cell types. We stress again that as target genes we considered those with a relative abundance in IP pellets, as compared to total RNA, greater than 2-fold. As a representative case of this normalization effect we refer here to the Gbp2 mRNA; its expression level was significantly affected in the HuR KO compared to HuR WT cells while it had the same degree of enrichment in the IP pellets of the two cell types (see Table S2 and sub-section 2.3.). If we had not normalized for its nuclear expression level, Gbp2 mRNA would have appeared, instead, with a reduced association in the HuR KO IP pellets as a result of its reduced expression.

**Figure 2:**
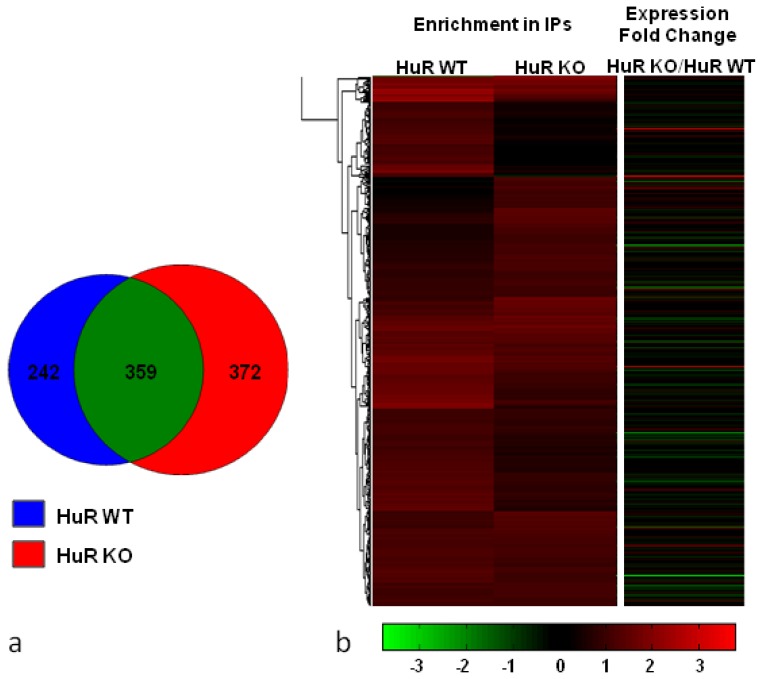
hnRNP A1-RNP-associated RNA targets in nuclear extracts of HuR WT and HuR KO MEFs (**a**) Venn Diagram representing the comparison of target genes between the two cell types following microarray data analysis; (**b**) Hierarchical clustering on the union of the identified RNA targets in HuR WT and HuR KO cells, depicting their differential association with hnRNP A1-RNP complexes in both cell types. Their expression fold change in total RNA is shown in a separate heat map on the right.

This first analysis of target genes for HuR WT and HuR KO cells is shown in the Venn diagram (Figure 2a) that indicated the presence of distinct groups, common and exclusive. We note that as common transcripts we considered those ascribed with an equal or differential association level in the hnRNP A1-RNPs of the two cell types. The percentage of the exclusive group of target genes was higher in HuR KO (49%) relative to HuR WT cells (40%).

In order to address the RNA targets related to HuR, we performed hierarchical clustering on the union of the identified hnRNP A1-RNP-associated transcripts in HuR WT and HuR KO cells (Figure 2b). This direct comparison showed that the bulk of identified transcripts had a relatively high enrichment level (>1 in log2 scale) in both cell types, indicative of their stable association with the isolated RNP complexes that was independent of the presence or absence of HuR. Clearly, however, there were two distinct groups of RNA transcripts affected by HuR (showing either increased or reduced association). In line with the data presented in the Venn diagram, there was a larger group of transcripts having increased association with the hnRNP A1-RNPs in HuR KO and reduced in HuR WT cells, compared to the reverse case.

The identity of all associated RNAs in HuR KO against HuR WT cells, based on the degree of their enrichment (in the range between 4 to 0.1 fold changes), is presented in Table S2. For the sum of 973 target genes in both cell types, an estimated number of 670 (over 50%) were in the range of 1.40 to 0.70 and were considered as RNA transcripts practically non-affected by the absence of HuR. Furthermore, 336 and 160 transcripts had a fold change in their enrichment over 1.40 or below 0.70 and were taken to represent target genes with increased or reduced association, respectively. This was again in line with the data on the hierarchical clustering presented in Figure 2b, showing a larger fraction of RNA transcripts with increased association in the HuR-lacking compared to HuR-containing hnRNP A1-RNP complexes.

In an effort to further investigate the influence of HuR’s presence on the regulation of the target genes, we compared the list of differentially expressed transcripts in total RNA presented in Table S1 to the one of hnRNP A1-RNP-associated targets presented in Table S2. Only 29 common genes were found. Even when relaxing the statistical criteria while retaining the expression fold change cut off at the level of 2-fold, the number of common genes remains small, as only 88 genes characterized as hnRNP A1-RNP targets displayed a significant alteration at the level of gene expression (Table S3). For a more detailed bioinformatic analysis, we provide for the entire list of the identified targets their expression fold change in the total nuclear RNA population of HuR KO *vs*. HuR WT cells, represented by the separate heat map in Figure 2b. By all means of comparison, we deduced that only a small fraction (<10%) of the target genes associated with the IP pellets of HuR KO and HuR WT cells were affected by the absence of HuR at the level of gene expression in the total nuclear RNA population.

For the purpose of gaining further insight concerning the biological functionalities of RNA transcripts in association with the immunopurified hnRNP A1-RNP complexes in HuR WT and HuR KO cells, the list of identified targets were subjected to GO analysis focused on the GO category of “Biological Processes” (BPs). The GOs ascribed to the nuclear hnRNP A1-RNP-associated target genes separately for the HuR WT and HuR KO cells are given in Table 3. A larger number of GOs were provided for the RNP-associated targets compared to those of differentially expressed transcripts in total nuclear RNA. Of special interest was the finding that most of the annotated nuclear transcripts appeared to affect BPs relevant to those anticipated for hnRNP A1 and HuR biological functioning [1,2,13,24]. With respect to HuR WT cells, we note the prominent presence of BPs related to post-transcriptional (such as translation, RNA splicing, mRNA processing, transport), as well as transcriptional events. In the case of HuR KO cells, of special interest was the appearance of unique cases of BPs, such as apoptosis, cell cycle, DNA repair, cell division and mitosis.

### 2.3. Validation by RT-qPCR of Selected mRNA Targets: Presence of Spliced and Pre-Spliced mRNAs in Isolated hnRNP A1-RNP Complexes

The validity of the microarray data in defining target genes associated with HuR-containing and HuR-lacking hnRNP A1-RNP complexes was tested by RT-qPCR. We selected for validation 8 target genes with likely relevance to hnRNP A1 and/or HuR biological function shown in Table 4. These referred to transcription and RNA processing factors, genes involved in DNA repair, immune response, cell cycle regulation and development. The mRNAs of the target genes selected for validation had a differential hnRNP A1-RNP association level (either increased or reduced) or a minor change in HuR KO/HuR WT cells, as depicted in Table 4 by their ratio of enrichment (see also Table S2).

**Figure 3:**
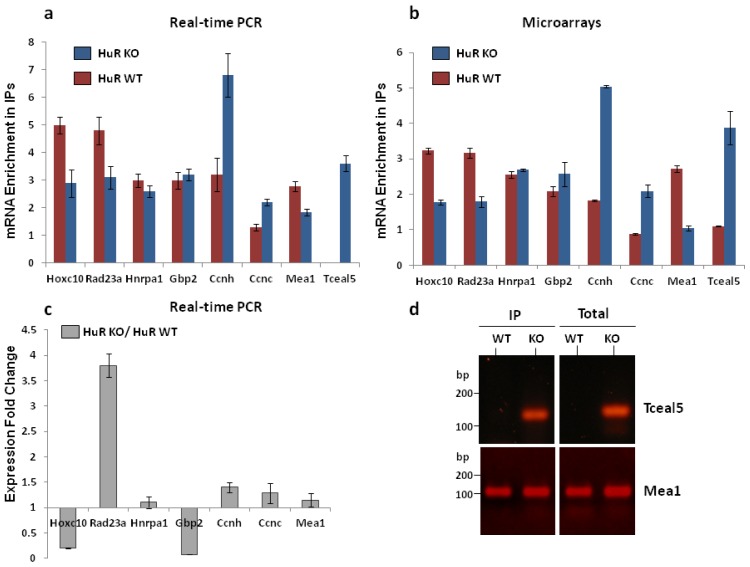
Validation of RIP-Chip data (**a**) RT-qPCR on RNA isolated from hnRNP A1 IPs and total nuclear extracts was performed to calculate the enrichment of mRNA for 8 target genes in HuR WT and HuR KO cells, namely *Hoxc10*, *Rad23a*, *HnRNP A1*, *Gbp2*, *Ccnh*, *Mea1* and *Tceal5* and compared to the microarray analysis; (**b**) Gapdh mRNA was used for normalization. The signal for Tceal5 mRNA transcript in HuR WT cells remained at the background level. Values represent the mean of three independent experiments performed in triplicates and error bars the standard deviations; (**c**) Fold change in expression of the selected genes in HuR KO as compared to HuR WT cells as determined by RT-qPCR on total nuclear RNA from the two cell types. Values and error bars are as described above. Genes with an expression fold change <1 correspond to down-regulated genes and are graphically shown in the opposite direction; (**d**) Agarose gel electrophoresis of the products of two RT-qPCR reactions, namely for Tceal5 and Mea1 transcripts, indicating that Tceal5 mRNA was undetected in HuR WT cells even after extension of the reaction cycles. The amplicon corresponding to Mea1 is shown as a positive control.

In the RT-qPCR reaction, the gene specific pair of primers (File S1) was designed to span an exon-exon junction and to yield the spliced product, alone. The RNA obtained from the triplicate experiments on HuR WT and HuR KO cells was used to estimate the actual mRNA levels of the selected target genes both in the total nuclear RNA population and in the RNA fraction associated with the hnRNP A1-RNP complexes (IPs). The level of Gapdh mRNA was used as an internal non-target normalization index in the qPCR reaction. On the basis of the estimated mRNA enrichment in IP reactions by RT-qPCR (Figure 3a), as well as by microarray analysis (Figure 3b), a good correspondence could be inferred for the validated genes. In both cases, the enrichment of hnRNP A1 mRNA targets in the RNP complexes was obtained in relevance to the expression level of the validated gene in the total nuclear RNA and they thus reflected upon any change in their association state. In sum, six genes showed an increased (*Ccnh*, *Tceal5*, *Ccnc*) or reduced (*Hoxc10*, *Rad23a*, *Mea1*) association with the HuR-lacking hnRNP A1-RNP complexes, while two (*hnRNP A1*, *Gbp2*) had minor changes in their association state in the two cell types. In addition, an indication of the changing ratio in the expression level of the validated mRNAs in total nuclear RNA in HuR KO *vs*. HuR WT cell is presented (Figure 3c). As seen, three mRNAs had major changes in their expression level, either up-regulated (Rad23α) or down-regulated (Hoxc10 and Gbp2), while the rest had a relatively slight increase in HuR KO. The direct comparison between the hnRNP A1-RNP-associated mRNAs and their expression level in the nuclear RNA population brought up some interesting findings. In HuR WT, in contrast to HuR KO, cells the Tceal5 mRNA was considered a non-target transcript (below the baseline level) by microarray analysis and was, also, totally undetected by RT-qPCR (both in the total nuclear RNA and the IP pellets) (Figure 3d). A first indication of the low expression of *Tceal5* gene in HuR WT cells was its detected p-value (>0.01) based on the microarray data, suggesting that it could be characterized as absent. However, Tceal5 mRNA was clearly identified in HuR KO cells and, moreover, had a high affinity for the respective hnRNP A1-RNP complexes (Figure 3a,b). Another case of interest was that of Rad23α mRNA, that while highly induced in the nuclear RNA of HuR KO cells, had a reduced affinity for the corresponding RNP complexes. Of the two mRNAs down-regulated in the HuR KO nuclear RNAs, Hoxc10 had a parallel significant reduction in the isolated hnRNP A1-RNPs while the affinity of Gbp2 mRNA, as also mentioned above (sub-section 2.2.), was practically unaffected. Of the remainder validated genes with a relatively small change in their nuclear mRNA levels between the two cell types (*Ccnh*, *Ccnc* and *hnRNP A1*), we point to a disproportional increase in Ccnh mRNA affinity for the HuR-lacking RNP complexes (compare histograms in Figure 3a–c).

**Figure 4:**
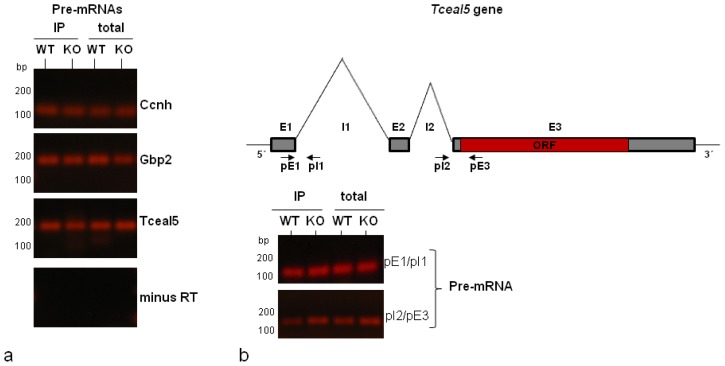
Detection of pre-mRNAs of Tceal5, Gbp2 and Ccnh in hnRNP A1 IPs. (**a**) RT-PCR using primers specific for the unspliced transcripts were used to detect the pre-mRNA forms of the tested genes, as identified both in the total nuclear RNA and in the IPs of HuR WT and HuR KO cells. Control reactions in the absence of reverse transcriptase, to exclude the amplification of contaminated DNA, are also shown; (**b**) Schematic representation of the *Tceal5* gene organization, showing the position of the primers used to amplify the Tceal5 pre-mRNA. RT-qPCR verification, using two different pairs of primers, of the Tceal5 pre-mRNA in IPs and total nuclear RNA in both cell types (in contrast to the spliced transcript detected only in HuR KO cells; Figure 3d).

The early loading of the hnRNP proteins on the nascent RNA pol II transcripts of pre-spliced mRNAs has been well documented [1,2]. This is also the case of HuR that binds pre-mRNA forms [37] and, as inferred from our previous findings, has the ability to associate (in addition to RNA processed low-salt-released nuclear hnRNPs) with the nuclear matrix-bound and chromatin-released hnRNP A1-RNP complexes; the latter anticipated to represent more nascent transcripts [31]. We, thus, searched for the presence, within the immunoselected nuclear hnRNP A1-RNPs of the pre-mRNA forms corresponding to three of the validated mRNA transcripts (Tceal5, Ccnh and Gbp2). RT-qPCR assays were repeated on the nuclear RNA population and the immunoprecipitated RNAs of HuR WT and HuR KO cells by designing pairs of RNA primers (File S1) so that one primer per pair aligned within an exon and the other in the neighboring intron to amplify the unspliced, pre-mRNA product. As seen in Figure 4a, the pre-mRNA form of the tested genes was clearly identified, in the RNA pool and the IP pellets of both cell types. Control reactions without reverse transcriptase were performed to exclude the amplification of contaminated DNA. This finding, therefore, indicated the integrity and stability of our immunopurified nuclear hnRNP A1-RNP complexes by their ability to sustain binding of both pre- and spliced mRNAs.

An unexpected finding of the above assay was the detection of the Tceal5 pre-mRNA in both HuR-lacking and HuR-containing cells, in clear opposition to the absence of its mature mRNA in HuR WT cells (compare Figures 3c and 4a). The schematic diagram of Figure 4b, outlines the *Tceal5* gene organization consisting of three exons (E1–E3). Exons E1–E2 and the two intervening sequences (I1 and I2) are located at the 5′ end, while exon E3 contains the open reading frame (ORF) and the 3′UTR of the gene. In the spliced mRNA, the joined E1–E2 exons form the major part of a 5′UTR region. The pair of primers pE1/pI1 that corresponded to the first exon (E1) and intron (I1) was initially used to detect the Tceal5 pre-mRNA product shown in Figure 4a. Moreover, the application of a second pair of primers located in intron I2 and exon E3 (pI2/pE3) was able (as in the case of the pE1/pI1 pair) to amplify a Tceal5 pre-mRNA product, as shown in Figure 4b upon the parallel application of both pair of primers. Based on the above, we have confirmed the association of Tceal5 pre-mRNA with the hnRNP A1-RNP complexes in both HuR WT and HuR KO, with the spliced mRNA being unique to the HuR KO cells. The simplest way to interpret this interesting finding is to hypothesize a post-transcriptional regulatory mechanism operating on the *Tceal5* gene, at the level of pre-mRNA splicing.

## 3. Discussion

Our present study is the first report on the application of a holistic approach to identify the HuR-dependent pattern of hnRNP A1-RNP-associated RNA transcripts, using Ribonomics on HuR-containing and HuR-lacking MEF cells. By taking into account that the bulk of cellular HuR, as for most of hnRNPs, resides in the nucleus [19,20] and that the cytoplasmic role of HuR has been, so far, mostly scrutinized [13,17], we focused here on mapping the sub-population of mRNA transcripts in stable association with nuclear hnRNP A1-RNP complexes in the presence or absence of co-precipitated HuR.

Several reports already exist on the application of the RIP-Chip platform on immunoselected either hnRNP- [36] or HuR- [38,39] RNP complexes (as well as hnRNP- and HuR- RNP assemblies [37,40]), in a number of cellular systems and under different growth states. Since so far, the hnRNP A1 as well as HuR mRNA targets have been tested on RNAs obtained from whole-cell lysates or cytoplasmic extract of a variety of mammalian cell types [36,37,39], our study is unique in this aspect as it focused on the nuclear compartment, alone. The most relevant to our work report is that of Katsanou *et al*., [33] that provided a list of mRNAs with a differential expression in whole cell extracts obtained from HuR WT and HuR KO MEF cells. Having used in the present study the same MEF cells, we stress again the novelty of our approach to determine the sub-population of HuR-dependent RNA transcripts associated with nuclear hnRNP A1-RNP complexes. Considering the fact that in the above report [33] the RNA population subjected to microarray analysis was obtained from a whole cell extract, which is anticipated to be mostly enriched in the cytoplasmic mRNA pool, we performed a comparison between the lists of significantly differentiated genes reported in [33] and our present study (Table S1). The number of common genes was limited, as only 22 genes exhibited altered expression in both studies. This could be due to the aforementioned different origin of the examined RNA, but also to the different cut offs applied in each study. For this reason, we applied the lower fold change cut off of 1.5 used by Katsanou *et al*. [33] (instead of 2) and less strict statistical criteria; the number of genes exhibiting altered expression in both studies raised to 102, which corresponds to 28% of the differentiated genes reported in [33].

The Biological Processes affected by the absence of HuR, as indicated by the GO analysis of these genes, mainly relate to development, regulation of transcription and immune response.

As our main effort was to define the RNA components of hnRNP A1-RNP complexes from HuR WT and KO cells, we have not looked into any possible alterations in the protein composition of the isolated complexes. Nonetheless, we have commented in the Results on the apparent absence of major changes in the nuclear level of hnRNP A1 in the HuR-containing *vs*. HuR-lacking cells. An indirect support of the above claim was also provided by the microarray-based quantitative estimates of hnRNP A1 mRNA itself, as similar amounts were found between HuR WT and HuR KO nuclear extracts and in the respective IP pellets. The existence of other type of translational as well as post-translational (e.g., protein phosphorylation or methylation) effects cannot be excluded. We also inferred from Figure 1 that a relatively small fraction of the nuclear HuR protein was associated in an RNA-dependent manner with the isolated hnRNP A1-RNP complexes. HuR (and hnRNP A1 alike) are well known multifunctional proteins participating in several macromolecular assemblies by forming protein-RNA as well as protein-protein interactions [2,13]. As also illustrated by RIP-Chip and iCLIP studies [22,41,42], the HuR RNA targets represent a large portion of the transcriptome (about 15%). We conclude from the above that the HuR-dependent RNA targets of the hnRNP A1-RNP assemblies reported in the present work represent a minor sub-fraction of the total HuR-bound nuclear targets.

With respect to the differential gene expression in HuR KO *vs*. HuR WT nuclear extracts, special reference is made to the genes up-regulated in HuR KO with a role in transcription, proteasome assembly, cell signaling and myogenesis. In the down-regulated RNA transcripts we note the high ranking of matrix metallopeptidases and ubiquitin specific peptidases functioning in the degradation of extracellular matrix and progression of inflammatory responses, as well as the members of the cytochrome P450 family having a role in detoxification. After normalizing for the expression level of RNA transcripts in the nuclear pool of HuR WT and HuR KO cells, the nature of the mRNA targets and their degree of enrichment in the hnRNP A1-RNPs of either cell type have been clearly linked to their affinity for the isolated RNP complexes. As seen in the priority list of the top HuR WT and HuR KO RNA targets (Table 2), only the AU RNA binding protein coenzyme A hydratase exhibited high affinity (fold change enrichment >4) in both HuR WT and HuR KO cells. The great majority of the targets had a high enrichment in HuR KO cells and included genes relevant to the discrete GOs uniquely seen in the HuR KO cells, with special reference to those affecting the ubiquitin-dependent protein catabolic process, apoptosis, cell cycle, DNA repair, cell division and mitosis. It is worth noting here a recent study [43] where the GO analysis of HuR-interacting transcripts revealed an unexpected striking enrichment for members of the ubiquitin ligase conjugation pathway that led the authors to suggest a new role of HuR in the regulation of protein degradation. Regarding DNA repair, it is worthwhile mentioning that Rad23a, a critical enzyme in Nucleotide Excision Repair [44], and Ccnh, a major component of TFIIH in Transcription Coupled Repair [45], are mRNAs targets strongly enriched in the hnRNP A1-RNP complexes of HuR KO cells. These findings are in line with the emerging role of RNA-binding proteins, including HuR and members of the hnRNPs (hnRNP B1, C1/C2, K), in the cellular response to genotoxic stress via modulation of the capacity of the DNA repair enzymes [46–49] and they thus deserve further investigation.

By all relevant reports, HuR and hnRNP proteins can simultaneously associate on common nuclear as well as cytoplasmic RNPs, through their joined binding (via RNA-dependent associations) on non-overlapping sites of the target RNA [37,50]. Along these lines are our current findings that amongst the target genes bound to nuclear hnRNP A1-RNP complexes in the HuR WT MEF cells, we have identified many known mRNA targets (by themselves or as members belonging to the same family) of either hnRNP A1 or HuR. We refer here to the hnRNP A1-associated mRNAs Mki67, serpine1, Whsc1, Tax1bp1, as well as the hnRNP A1 mRNA itself, a known hnRNP A1 mRNA target [36,51] in human keratinocytes Colo 16 cells [10] and K562 cells [36]. Among the reported HuR-associated targets are members of the Rad, Cdk and Cd family [37,52]. Based on this small group of examined transcripts, the validity of our experimental approach to ascribe the sum of the identified mRNAs as true targets of nuclear HuR-containing hnRNP A1-RNP particles in MEF cells has gained additional support.

The absence of HuR had an overall greater impact on the hnRNP A1-RNP-bound RNA compared to its presence as supported by all findings (see also Venn diagram and Heat map in Figure 2). Currently, a possible way to interpret these findings would be that the lack of hnRNP A1-RNP-associated HuR may affect the turnover and overall RNA processing of a number of bound transcripts, and consequently lead to their nuclear retention. Given the recently strengthened participation of HuR in nuclear functions, involving splicing, polyadenylation [21,22,42], as well as its implicated role in mRNA export [53–55], we consider the possibility of a blockade or reduced rate in the nuclear processing for a subset of HuR-dependent pre-mRNA targets that remained bound to hnRNP A1-RNPs in the absence of HuR and do not exit the nucleus. In support of the above, the splicing of pre-mRNA is known to be coupled with mRNA export and defects in splicing can lead to nuclear sequestration and mRNA decay [53,56]. Thus, in the HuR KO cells nuclear retention of specific mRNA cargoes could take place. Considering the many alternate functions of HuR, and not only its stability/translational activity in the cytoplasmic compartment, defects in pre-mRNA splicing and/or polyadenylation events or changes in ARE-mediated properties might be involved in altering mRNA export activity of HuR KO cells. Overall, the recognition of a significant change in the associated RNA transcripts (Table S2) is suggestive of a dynamic composition of hnRNP A1-RNPs in relation to HuR’s presence that is expected to affect, directly or indirectly, the many cellular functions regulated by HuR. We should also consider at this point the possibility that this rather drastic reprogramming of gene expression in the absence of HuR may be related, to a greater or lesser degree, to the cellular adaptation rather than being related directly to HuR deficiency. As this pivotal study opens new fields of analysis worth investigated in future studies, we believe that such relevant questions can be experimentally approached.

In addition to the cytoplasmic functioning of HuR in regulating the stability, translation or both of the bound mRNAs [15,16], its potential for a greater than initially anticipated nuclear impact in post-transcriptional regulatory processes is currently gaining a lot of attention. Based on studies with individual mRNAs, HuR has been initially implicated in regulating alternative pre-mRNA splicing and cleavage/polyadenylation events [13,21,42,57,58]. At a transcriptome-wide level, the recently applied combined methods of PAR-CLIP and RIP-Chip assays unraveled a surprisingly large number of RNA-HuR interactions occurring in introns (1/3 of all associations) with many of them in the proximity of 3′ splice sites [22,41,42]. Direct comparison of the two methods showed an extensive overlap of the identified HuR targets and, furthermore, led to the conclusion that the RIP-Chip method can indeed identify authentic targets of cellular HuR.

We have commented in the Results on the detection of the pre-mRNA version for few RT-qPCR validated mRNA transcripts within the immunoselected hnRNP A1-RNPs. This indicates that not only the processed mRNAs but also their pre-spliced forms have an affinity for the isolated hnRNP A1-RNP assemblies from the nuclear extracts. A particular, distinct case in support of a direct or indirect (via another interacting component) involvement of HuR in nuclear mRNA processing was that of the Tceal5 mRNA transcript, a member of the Tceal family functioning as RNA polII elongation factors. As also outlined in the Results, Tceal5 pre-mRNA, although associated with hnRNP A1-RNP complexes in both HuR WT and HuR KO cells, did not process to mature mRNA in the presence of HuR. Most likely, removal of HuR from the RNP complexes released this blockage, which suggests a post-transcriptional regulatory mechanism of HuR via competition with hnRNP proteins, possibly at the level of splicing. Similar to Tceal5, additional cases of HuR’s participation in the regulation of gene expression at the post-transcriptional level might be unraveled in future studies.

Finally, in our present study we have chosen to work on two essential multifunctional RBP proteins; HuR a known major modulator in inflammation as well as cancer and hnRNP A1 a prototype hnRNP protein involved in splicing (mainly alternative splicing) that also exhibits altered expression in carcinogenesis. We believe that expanding the current study on MEF cells to include other cell types, such as macrophages and epithelial cell cultures, could possibly provide for new biological markers for disease diagnosis and prognosis, as well as novel molecular targets for therapeutic intervention.

## 4. Experimental Section

### 4.1. Experimental Cellular System

The initial construction of the HuR knockout (KO) mouse embryos and the isolation of Mouse Embryonic Fibroblast (MEF) cells from the E12.5 stage of either HuR WT or HuR KO embryos has been described before, as is their maintenance in cell culture [33]. HuR WT and HuR KO MEF cells were the kind gift of DL Kontoyiannis (Dept. of Immunology, Alexander Fleming Biomedical Sciences Research Centre, Vari, Greece) and were maintained in exponential growth in D-MEM medium supplemented with 15% heat-inactivated FCS. The main phenotype of the HuR KO MEF cell culture, compared to the HuR WT, was their reduced growth rate (over twice the generation doubling time of HuR WT cells) and the requirement of plating at higher cell density upon sub-culturing.

### 4.2. Nuclear Extract Preparation

Nuclear extracts were obtained from MEF cells using the classical protocol of Dreyfuss *et al*. [34] that is also known to preserve the integrity of isolated RNP complexes. Specifically, a frozen cell pellet of about 3 × 10^7^ cells was resuspended in 750 μL buffer A (10 mM Tris-HCl, pH 7.5, 100 mM NaCl, 2.5 mM MgCl) in the presence of 1 mM PMSF and 0.5% Triton X-100. The cell suspension was allowed to stay on ice for 5 min with gentle shaking and cell lysis followed by homogenization in a glass homogenizer with 10 strokes, on ice. The homogenate was centrifuged at 4000 rpm, 10 min at 4 °C in an Eppendorf centrifuge and the supernatant (representing the cytoplasmic extract) was removed. The nuclear pellet was, thereafter, resuspended in half the volume (350 μL) of buffer A without Triton X-100 and with the addition of 2 μg/mL protease inhibitor mix (leupeptin, pepstatin, aprotinin) (Sigma-Aldrich, St. Louis, MO, USA). After sonication (2 × 5 s) on ice, the resulted lysate was layered on top of two volumes of a 30% sucrose cushion in the same buffer used for nuclei suspension and centrifuged at 5000 rpm, 15 min at 4 °C. The new supernatant (nuclear extract) was carefully collected from the top of the sucrose cushion and immediately frozen in liquid nitrogen. A similar extraction protocol was performed on triplicate experiments for HuR WT and HuR KO cells, alike. Protein estimates with the BioRad reagent were performed on a small aliquot of the nuclear extract. The total nuclear protein corresponding to the 3 × 10^7^ cell pellet of either HuR WT or HuR KO cultures was about 2.5–3.0 mg.

### 4.3. RNA Immunoprecipitation Coupled with Microarray Analysis—RIP-Chip Assay

#### 4.3.1. RNA Immunoprecipitation (RIP) Step

Fractionated nuclear extracts from the triplicate experiments (each containing about 3 × 10^7^ cells) were subjected to immunoprecipitation (IP) assays applying the basic procedure used before [31] slightly modified according to the protocol of Baroni *et al*. [59]. All steps took place at 0 to 4 °C, starting with the preparation of antibody coated protein A-sepharose (PAS) bead matrix (Sigma-Aldrich, St. Louis, MO, USA). 10 μg of either the mouse monoclonal anti-hnRNP A1 (4B10) antibody (Santa Cruz Biotechnology, Santa Cruz, CA, USA), or the mouse IgG2 purified immunoglobulin of same isotype as the anti-hnRNP A1 antibody that served as control IP was incubated with 50 μL packed volume of PAS in 1 mL buffer A, overnight at 4 °C with gentle rocking, followed by extensive washing of the beads (4× in 1 mL buffer A) and short spinning at 4000 rpm in an Eppendorf centrifuge (Eppendorf, Hauppauge, NY, USA). Thereafter, the quickly thawed nuclear extracts were first clarified at 13,000 rpm, 10 min to remove any insoluble material and, then, brought to a final volume of 1 mL in buffer A with the addition of PMSF, EDTA, DTT, RNase out and placental RNase inhibitor at the amounts indicated in Baroni *et al*. [59]. Before IP, pre-clearing of the nuclear extracts was performed to reduce unspecific binding by 30 min incubation with PAS beads been pre-coated with 6 μg IgG2. After a quick spin to discard beads, extracts were transferred to a clean tube and a 100 μL (1/10th) aliquot removed and stored for subsequent RNA extraction. The remainder (900 μL) of the extract was mixed with the pre-coated PAS-IgG2 bead matrix and incubated for 2 h at 4 °C, with rocking. The PAS-IgG2 pellets were collected by a quick spin and kept on ice, while the resulted supernatant was transferred to the matrix of the anti-hnRNP A1 beads and incubated for an additional period of 2 h at 4 °C. After a final spin to obtain the anti-hnRNP A1 immune pellets, all IPs (including those of control IgG2) were extensively washed in NT2 buffer (50 mM Tris-HCl, pH 7.4, 150 mM NaCl, 1 mM MgCl_2_, 0.05% NP-40) with 1 mM PMSF, 5 times with a 5 min interval in between.

#### 4.3.2. RNA Extraction Step

The immune pellets (IPs) and stored nuclear aliquots were subjected to RNA extraction. First, the washed immune pellets were resuspended in 100 μL NT2 buffer and 1 mM PMSF and an aliquot (1/20th) removed and stored for protein resolution and western blotting. Similarly, an aliquot corresponding to 1/50th of the total extract applied per IP reaction was also stored (see immunoblotting). The remaining main portion of immune pellets and nuclear extracts were subjected to proteinase K digestion, quickly spun to remove the empty beads, and processed through the RNA extraction protocol using the RNeasy Mini Kit column (Qiagen, Duesseldorf, Germany) following the manufacturer’s instructions. On-column DNA digestion (RNase-Free DNase Set, Qiagen, Duesseldorf, Germany) was used to ensure the absence of DNA from the samples. The quantification and quality analysis of RNA was performed on a Bioanalyzer 2100 (Agilent, Santa Clara, CA, USA).

#### 4.3.3. Microarray Hybridization and Data Analysis

Synthesis of cDNA and biotinylated cRNA was performed with the Illumina TotalPrep RNA Amplification Kit (Illumina, San Diego, CA, USA) using 500 ng of total or immunoprecipitated RNA. Hybridization was performed onto Illumina MouseWG-6_V2_Expression BeadChips (Illumina, San Diego, CA, USA) according to manufacturer’s instructions. Three biological replicates were used for each condition. The raw data were analyzed using GeneArmada software [60]. Briefly, background corrected values were normalized by Quantile method and log2 transformed. Probesets with a detection *p*-value > 0.01 in all tested conditions were not considered. Significantly enriched probe sets in IP samples as compared to total nuclear RNA in HuR KO and HuR WT cells were identified by *t*-test and characterized as hnRNP A1-RNP targets by applying the following thresholds: *p-*value < 0.05, False Discovery Rate (FDR) < 0.05 and a fold change >2. Similarly significantly differentiated genes in total nuclear RNA of HuR KO as compared to HuR WT cells were identified by *t*-test with the aforementioned thresholds. Probe sets not annotated with a RNA RefSeq were not considered for further analysis. The mouse platform contained 45281 probes corresponding to 30,855 different RNAs, mostly mRNAs but also a low percentage of non-coding RNAs. This is expected to provide whole-genome transcript coverage. GO-based analysis was performed with the web application StRAnGER [61] on the branch of GO concerning Biological Processes. The *p*-value cutoff of the hypergeometric test was set at 0.01.

### 4.4. Immunoblotting

Protein analysis was performed on an aliquot of nuclear extracts and immune pellets. Samples were directly mixed with SDS sample buffer and subjected to SDS-PAGE (10% polyacrylamide gel). Following transfer to nitrocellulose, the immunoblotting protocol used to detect the antigen-antibody reactions on the membrane was applied as before [31].

### 4.5. Real-Time PCR Analysis (RT-qPCR)

For the real time PCR assays on selected mRNA transcripts, 250 ng RNA obtained from the immunopurified hnRNP A1-RNP complexes or total nuclear RNA was reverse-transcribed using Superscript III (Invitrogen, Carsland, CA, USA) reverse transcriptase and an oligo-dT primer according to the manufacturer’s instructions. In the case of pre-mRNA targets the reverse transcription step was performed with random hexamer primers. Real-time PCR reaction products were synthesized with the application of appropriate primer pairs and quantified by the incorporation of SYBR Green I (iQ SYBR Green Supermix, Biorad, Hercules, CA, USA) on an iQ5 Real-Time PCR Detection System (Biorad, Hercules, CA, USA) using Gapdh mRNA as an internal control for normalization. All assays were performed in triplicate in a 25 μL reaction. Specificity of the amplified PCR product was assessed through a melting curve analysis and agarose gel electrophoresis of a small aliquot of the reaction and staining with ethidium bromide. For each qPCR reaction set a standard curve was obtained using serial dilutions of total nuclear RNA and calculations were performed according to the Relative Standard Curve Method as in Applied Biosystems technical notes (Guide to Performing Relative Quantitation of Gene Expression Using Real-Time Quantitative PCR).

The list of synthesized primers that were applied in qPCR reactions are provided in File S1. For mRNAs one primer per pair was designed to span an exon-exon junction, while for pre-mRNAs one primer was designed to anneal to an exon and its pair to an intron.

## 5. Conclusions

Our present study aimed at investigating the nuclear role of HuR in the context of its association with endogenous hnRNP complexes. To this extent, we report here on the novel application of the Ribonomics (RIP-Chip) platform on hnRNP A1-RNP complexes immunoselected from the nuclear extracts of HuR-lacking and HuR-containing mouse embryonic fibroblast (MEF) cells. This led to the identification of HuR-dependent RNA transcripts in stable association with nuclear hnRNP A1-RNP complexes as a specific sub-fraction of the total HuR-bound nuclear targets. Amongst the identified RNA transcripts were some known hnRNP A1 as well as HuR mRNA targets, proving the validity of our experimental approach. The nature and degree of enrichment of the detected RNA targets in the immunopurified hnRNP A1-RNPs have been linked to their affinity for the isolated RNP complexes by normalizing for their expression level in the nuclear pool of either HuR WT or HuR KO cells. All findings, in particular those that were based on the presence of specific GO categories and on the RT-qPCR validated mRNA targets (including some unspliced pre-mRNA forms), led to the recognition of a significant change in the HuR-lacking (KO) RNA transcript population, compared to HuR-containing (WT) cells. This reflected upon a dynamic composition of hnRNP A1-RNPs in relation to HuR’s presence that is likely to affect, to a greater or lesser degree, the many anticipated nuclear functions regulated by HuR.

## Figures and Tables

**Table 1:** Top up- and down-regulated genes in HuR KO *vs*. HuR WT MEFs as indicated by microarray analysis on total nuclear RNA. Expression-fold changes and relevant *p*-values following *t*-test analysis are shown.

	Gene Symbol	Definition	*p*-value	Fold increase/decrease
**Up-regulated genes**	*Pitx2*	paired-like homeodomain transcription factor 2, transcript variant 2	0.00503	90
	*Psmd8*	proteasome (prosome, macropain) 26S subunit, non-ATPase, 8	0.00007	53
	*Gm15698*	predicted gene, transcription elongation factor B (SIII), polypeptide 2, non-coding RNA.	0.00363	23
	*Glrx1*	Glutaredoxin	0.00080	21
	*Parm1*	prostate androgen-regulated mucin-like protein 1	0.00021	21
	*Gfpt1*	glutamine fructose-6-phosphate transaminase 1	0.00050	17
	*Csprs*	cytokine receptor-like factor 1	0.00041	15
	*Thumpd1*	THUMP domain containing 1	0.00002	13
	*Sorbs2*	sorbin and SH3 domain containing 2	0.00298	13
	*Mef2c*	myocyte enhancer factor 2C	0.00551	12

**Down-regulated genes**	*Xlr4a*	X-linked lymphocyte-regulated 4A	0.00200	81
	*Mmp3*	Matrix metallopeptidase 3	0.00006	71
	*Cyp7b1*	cytochrome P450, family 7, subfamily b, polypeptide 1	0.00143	69
	*Rsad2*	radical S-adenosyl methionine domain containing 2	0.00161	49
	*Osr2*	odd-skipped related 2 (Drosophila)	0.00032	48
	*Tomm22*	translocase of outer mitochondrial membrane 22 homolog (yeast), nuclear gene encoding mitochondrial protein	0.00012	42
	*Atf4*	Activating transcription factor 4	0.00098	32
	*Mmp13*	matrix metallopeptidase 13	0.00397	31
	*Usp18*	ubiquitin specific peptidase 18	0.00562	24
	*Mmp10*	matrix metallopeptidase 10	0.00190	23

**Table 2:** List of the most enriched hnRNP A1-RNP-associated transcripts (fold change enrichment >4) in HuR WT and HuR KO MEFs.

	Gene Symbol	Definition	*p*-value	Enrichment
**Top Targets in HuR WT**	*Ppp1r11*	protein phosphatase 1, regulatory (inhibitor) subunit 11	0.00041	4.90
	*Pdcd5*	programmed cell death 5	0.00412	4.78
	*Auh*	AU RNA binding protein/enoyl-coenzyme A hydratase	0.00822	4.26

**Top Targets in HuR KO**	*Serf1*	small EDRK-rich factor 1	0.00029	5.52
	*Gm4832*	predicted gene Gm4832	0.00018	5.38
	*Dynlt3*	dynein light chain Tctex-type 3	0.00361	5.18
	*Ccnh*	cyclin H	0.00072	5.03
	*Spp1*	secreted phosphoprotein 1	0.00006	5.01
	*Uchl5*	ubiquitin carboxyl-terminal esterase L5	0.00004	4.97
	*Mrps18c*	mitochondrial ribosomal protein S18C	0.00002	4.84
	*Hspe1*	heat shock protein 1 (chaperonin 10)	0.00008	4.83
	*Ndufa2*	NADH dehydrogenase (ubiquinone) 1 alpha subcomplex, assembly factor 2	0.00047	4.81
	*Tnfaip8*	tumor necrosis factor, alpha-induced protein 8	0.00781	4.81
	*Etfa*	electron transferring flavoprotein, alpha polypeptide	0.00035	4.76
	*Rps3a*	ribosomal protein S3a	0.00271	4.66
	*Dazap2*	DAZ associated protein 2	0.00008	4.53
	*Cfdp1*	craniofacial development protein 1	0.00169	4.52
	*Rexo2*	REX2, RNA exonuclease 2 homolog (S. cerevisiae)	0.00016	4.45
	*Naca*	nascent polypeptide-associated complex alpha polypeptide	0.00021	4.41
	*Gnpda2*	Glucosamine-6-phosphate deaminase 2	0.00008	4.34
	*1810022K09Rik*	RIKEN cDNA 1810022K09 gene	0.00000	4.22
	*Auh*	AU RNA binding protein/enoyl-coenzyme A hydratase	0.00258	4.19
	*Hspa8*	Heat shock protein 8	0.00126	4.16
	*Cox20*	COX20 Cox2 chaperone	0.00121	4.04
	*Ppp1r11*	protein phosphatase 1, regulatory (inhibitor) subunit 11	0.00287	4.01

**Table 3:** Gene Ontology (GO) Analysis; the hnRNP A1-RNP targets identified in HuR WT and HuR KO MEFs were submitted to GO analysis, elucidating over-represented GO BP terms. *p*-value represents the hypergeometric test *p*-value score for each GO term. Enrichment represents the ratio of the number of times a GO term occurs in each list of target transcripts to the number of times this GO term exists in the list of the Illumina BeadChip.

	GO Annotation	*p*-value	Enrichment
**Targets in HuR WT**	Translation	0.00000000001	32/228
	protein transport	0.00000000039	26/435
	protein folding	0.00000000234	12/99
	intracellular protein transmembrane transport	0.00000000797	9/56
	RNA splicing	0.00000002600	14/166
	transport	0.00000007535	49/1500
	metabolic process	0.00000014388	26/574
	mRNA processing	0.00000014604	15/216
	transcription	0.00000115190	44/1412
	response to oxidative stress	0.00000167931	6/40
	oxidation reduction	0.00000416171	22/527
	protein catabolic process	0.00000510429	5/31
	regulation of transcription, DNA-dependent	0.00000672647	44/1512
	electron transport chain	0.00001006502	8/94
	translational initiation	0.00001094670	4/21

**Targets in HuR KO**	translation	0.00000000006	32/228
	transcription	0.00000000007	68/1412
	protein transport	0.00000000008	36/435
	regulation of transcription, DNA-dependent	0.00000000009	71/1512
	Ubiquitin-dependent protein catabolic process	0.00000000016	30/441
	apoptosis	0.00000003281	23/354
	transport	0.00000009805	56/1500
	cell cycle	0.00000053421	23/412
	vesicle-mediated transport	0.00000090952	12/134
	response to DNA damage stimulus	0.00000377796	13/176
	DNA repair	0.00000923405	12/165
	small GTPase mediated signal transduction	0.00004585070	12/192
	oxidation reduction	0.00009243521	22/527
	cell division	0.00040989564	11/209
	Mitosis	0.00095668335	8/140
	intracellular protein transport	0.00134751563	8/147
	mRNA processing	0.00184940082	10/216
	RNA splicing	0.00308169664	8/166
	metabolic process	0.00382078845	19/574

**Table 4:** List of mRNA targets selected for validation by RT-qPCR. The fold change in enrichments, observed in HuR KO as compared to HuR WT cells, is shown in the last column, where values close to 1 (1.4–0.7) indicate similar enrichments between the two cell types.

Gene Symbol	Definition	Features	Ratio of Enrichments KO/WT
*Tceal5*	transcription elongation factor A (SII)-like 5	Relief of transcription arrest by pol II; chromatin modification	3.52
*Ccnh*	cyclin H	Transcription-coupled repair; meiotic progression; embryonic development	2.77
*Gbp2*	guanylate binding protein 2	Member of GTPases; immune effector	1.23
*Hoxc10*	homeo box C10	Embryonic development; cellular transformation	0.55
*Rad23a*	RAD23a homolog (S. cerevisiae)	DNA damage response; proteasome degradation; oxidative stress	0.57
*HnRNP A1*	heterogeneous nuclear ribonucleoprotein A1	Prototype hnRNP protein; RNA processing (splicing and nuclear export)	1.84
*Ccnc*	cyclin c	Cell cycle progression and apoptosis	2.38
*Mea1*	Male-enhanced antigen 1	Mouse spermatogenesis	0.38
